# The timing of concurrent intrathecal chemotherapy during blinatumomab infusion influences neurotoxicity in pediatric acute lymphoblastic leukemia

**DOI:** 10.3389/fimmu.2025.1690916

**Published:** 2026-01-02

**Authors:** Bingju Liu, Yunpeng Dai, Liying Liu, Qi Wang, Guotao Guan

**Affiliations:** Department of Pediatrics, Shandong Provincial Hospital Affiliated to Shandong First Medical University, Jinan, China

**Keywords:** concurrent intrathecal (IT) chemotherapy, blinatumomab, neurotoxicity, pediatric, acute lymphoblastic leukemia

## Abstract

**Objective:**

To assess the safety profile of administering intrathecal (IT) chemotherapy concurrently with blinatumomab in pediatric patients with acute lymphoblastic leukemia (ALL).

**Methods:**

This retrospective analysis included 93 pediatric ALL patients treated between February 2021 and May 2025 who received blinatumomab.

**Results:**

Of the 93 enrolled patients (median [Q1, Q3] age, 6 [4, 12] years), 42 (45%) were given IT chemotherapy concurrently, while the remaining 51 (55%) served as controls. Neurotoxic events occurred in eight patients (8.8%) overall, with no statistically significant difference between the concurrent IT and control groups (12% vs. 6%, p = 0.461). However, receiving IT chemotherapy on Day 1 of blinatumomab was strongly associated with neurotoxicity (OR = 15.6; 95% CI: 2.96–87.8; p = 0.001). Additional univariate predictors included CD4+ T-cell count (OR = 0.03; 95% CI: 0.00–0.50; p = 0.045), serum albumin (OR = 1.17; 95% CI: 1.02–1.39; p = 0.042), and bone marrow blast percentage (OR = 1.05; 95% CI: 1.01–1.09; p = 0.017). Multivariate analysis identified Day 1 concurrent IT administration as an independent risk factor (OR = 12.5; 95% CI: 1.45–131; p = 0.023).

**Conclusions:**

Initiating IT chemotherapy on the same day as blinatumomab infusion significantly increases the risk of neurotoxicity in pediatric ALL patients.

## Introduction

Blinatumomab is a monoclonal bispecific T cell-engaging antibody that targets CD3 on T cells and CD19 on B cells, and it has become a key agent for the management of pediatric acute lymphoblastic leukemia (ALL) due to its high response rates and reduced hematologic toxicity compared to conventional chemotherapy. Despite these advantages, neurotoxicity remains a notable adverse event, consistent with other CD19-directed immunotherapies. Because blinatumomab has limited efficacy in treating extramedullary diseases, particularly in the central nervous system (CNS) (1), intrathecal (IT) chemotherapy continues to be a standard prophylactic and therapeutic measure in pediatric ALL. However, the safety of administering IT chemotherapy concurrently with blinatumomab, particularly with respect to the timing of administration, has not been fully investigated. This study was thus developed with the goal of evaluating the safety and timing of concurrent IT chemotherapy during blinatumomab therapy in a pediatric ALL cohort.

## Methods

This study was performed in accordance with the guidelines of the Declaration of Helsinki and was approved by the Institutional Review Board of the participating institution (No. 2025R022-E01). Written informed consent was obtained from the legal guardians of all participants. This retrospective study reviewed clinical records of 93 pediatric patients diagnosed with ALL without Down syndrome who received blinatumomab at our institution from February 2021 to May 2025. Patients with a low leukemic burden (bone marrow blasts <5%) were administered blinatumomab at 15 μg·m^−2^·day^−1^, while those patients with higher disease burden began at 5–10 μg·m^−2^·day^−1^ for 4–7 days before escalation to 15 μg·m^−2^·day^−1^. For patients with over 10% blasts in the bone marrow, blinatumomab was administered for 28 days, while for all others, a 14-day course was used. Dexamethasone (5 mg·m^−2^·day^−1^) was used as premedication prior to initiation, dose escalation, or post-interruption (≥4 hours) of blinatumomab. Forty-two patients received IT chemotherapy concurrently with blinatumomab, with such treatment being defined as receiving IT therapy either on the first day of blinatumomab (n = 4) or during the continuous infusion period (n = 38). All IT regimens consisted of triple therapy: methotrexate, cytarabine, and dexamethasone. The remaining 51 patients formed the control group, having received IT therapy outside the defined concurrent window. Neurotoxicity was evaluated using the Immune Effector Cell-Associated Encephalopathy (ICE) score and the Cornell Assessment of Pediatric Delirium (CAPD), as recommended by the American Society for Transplantation and Cellular Therapy (ASTCT) guidelines (2).

Statistical analysis of categorical variables was performed using chi-square or Fisher’s exact test, while continuous variables were assessed using Wilcoxon rank-sum or Student’s t-tests. Logistic regression analyses were conducted with R (v4.3.3) using the CBCgrps (v2.8.2), stats, and gtsummary (v2.0.3) packages.

## Results

Patient demographics and baseline characteristics are presented in **Table 1**. The cohort had a median age of 6 years (interquartile range (IQR): 4-12), and 67% were male. Of the 42 patients in the concurrent IT group, 14 (33.3%) received IT chemotherapy within 14 days of starting blinatumomab. Among these, four patients (28.6%) received IT therapy on the exact day of blinatumomab initiation, and the other 10 (71.4%) between Days 7 and 14. The remaining 28 patients (66.7%) received IT chemotherapy more than 2 weeks after starting blinatumomab. Baseline neuroimaging and neurological exams were normal in all patients prior to treatment. The cerebrospinal fluid (CSF) was analyzed in all pediatric cases that exhibited neurotoxicity; no abnormalities in cell counts, biochemical parameters, cytology, and flow cytometry were detected. The incidence of cytokine release syndrome (CRS) was 71.43% in the concurrent IT group compared to 64.71% in the control group; the difference between the two was not statistically significant. Neurotoxicity was observed in eight patients (8.8%), with no significant difference between the concurrent IT (12%) and control (6%) groups (p = 0.461). Median time to neurotoxicity onset was 3 days (IQR: 2–3) in the concurrent group and 5 days (IQR: 3–9) in controls (p = 0.349). Both electroencephalography (EEG) and cranial magnetic resonance imaging (MRI) were performed, with unremarkable results in all eight patients. Additionally, screening for Epstein–Barr virus, parvovirus B19, and cytomegalovirus was negative in all cases. Clinical symptoms included tremor, paresthesia, and seizures. Grade 4 neurotoxicity occurred in two of 42 patients (4.8%) in the concurrent IT group and one of 51 (2.0%) in the control group. Tremor was the most frequent symptom (concurrent IT: 11.9% vs. control: 2.0%; p = 0.087). Seven of the eight neurotoxicity cases occurred during full-dose blinatumomab (15 μg·m^−2^·day^−1^); one occurred during escalation (9 μg/day). Details of symptomatology and outcomes are provided in **Table 2**.

**Table 1:** Patient demographics and clinical outcomes.

Variables	Total (n = 93)	Control (n = 51)	Concurrent IT (n = 42)	P
Male sex, n (%)	62 (66.67%)	35 (68.63%)	27 (64.29%)	0.825
Age (years), median [Q1, Q3]	6 [4, 12]	6 [4, 11.5]	6 [5, 12.75]	0.414
IT within 2 weeks, n (%)	14 (15.05%)	0 (0)	14 (33.33%)	<0.001
IT on D1, n (%)	4 (4.30%)	0 (0)	4 (9.52%)	0.038
B cells, median [Q1, Q3]	0.01 [0, 0.19]	0.01 [0, 0.05]	0.01 [0, 0.25]	0.47
T cells, median [Q1, Q3]	1.13 [0.54, 1.66]	1.11 [0.53, 1.66]	1.14 [0.54, 1.64]	0.841
CD4+ T cells, median [Q1, Q3]	0.5 [0.24, 0.85]	0.53 [0.25, 0.96]	0.47 [0.23, 0.76]	0.317
CD8+ T cells, median [Q1, Q3]	0.51 [0.22, 0.86]	0.49 [0.23, 0.9]	0.51 [0.21, 0.81]	0.697
NK cells, median [Q1, Q3]	0.1 [0.05, 0.16]	0.11 [0.05, 0.17]	0.1 [0.05, 0.16]	0.969
B/T, median [Q1, Q3]	0.01 [0, 0.14]	0.01 [0, 0.09]	0.04 [0, 0.23]	0.297
WBC, median [Q1, Q3]	2.76 [1.9, 4.36]	2.83 [2.11, 4.48]	2.45 [1.41, 4.27]	0.175
Albumin level, median [Q1, Q3]	37.7 [33.4, 42.9]	38.1 [33.25, 43.1]	37.15 [33.62, 42.4]	0.796
Cycle of blinatumomab, median [Q1, Q3]	1 [1, 2]	1 [1, 2]	1 [1, 2]	0.673
Number of previous IT doses, median [Q1, Q3]	8 [2, 14]	9 [2, 17]	2 [2, 13]	0.152
History of CNS (+) ALL, n (%)	18 (19.35%)	7 (13.73%)	11 (26.19%)	0.211
Bone marrow blasts (%), median [Q1, Q3]	0 [0, 1%]	0 [0, 0.5%]	0.5 [0, 1.38%]	0.025
Relapsed or refractory ALL, n (%)	17 (18.28%)	11 (21.57%)	6 (14.29%)	0.526
All-grade neurotoxicity, n (%)	8 (8.60%)	3 (5.88%)	5 (11.90%)	0.461
Seizures, n (%)	3 (3.23%)	1 (1.96%)	2 (4.76%)	0.587
Tremors, n (%)	6 (6.45%)	1 (1.96%)	5 (11.90%)	0.087
Paresthesia, n (%)	2 (2.15%)	1 (1.96%)	1 (2.38%)	1
Time to neurotoxicity, median [Q1, Q3]	3 [2, 5]	5 [3, 9]	3 [2, 3]	0.349
Grade ≥ 3 neurotoxicity, n (%)	3 (3.23%)	1 (1.96%)	2 (4.76%)	0.587
Any grade CRS, n (%)	63 (67.74%)	33 (64.71%)	30 (71.43%)	0.64
Fever	62 (66.67%)	32 (62.7%)	30 (71.43%)	0.508
Anemia	28 (30.11%)	16 (31.37%)	12 (28.57%)	0.823
Neutropenia	33 (35.48%)	20 (39.22%)	13 (30.95%)	0.514
Thrombocytopenia	31 (33.33%)	18 (35.29%)	13 (30.95%)	0.825
Infections	5 (5.38%)	4 (7.84%)	1 (2.38%)	0.373
Low IgG	18 (19.35%)	10 (19.61%)	8 (19.05%)	1
Elevated transaminases	12 (12.90%)	9 (17.65%)	3 (7.14%)	0.214
Dose escalation, n (%)	55 (59.14%)	23 (45.10%)	32 (76.19%)	0.005

IT, intrathecal; B/T, B-cell count/T-cell count; WBC, white blood cell; CNS, central nervous system; ALL, acute lymphoblastic leukemia; CRS, cytokine release syndrome.

**Table 2:** Summary of patients who experienced neurotoxicity.

Patient characteristics			IT administration		Neurotoxicity symptoms, onset, and management					Other toxicities
No.	Age (y), sex	Bone marrow blasts	Preparation	BLIN day	Grade	Cycle of BLIN	NT symptoms (BLIN day)	BLIN dose	Interventions	
1	5, M	<1%	MTX 12 mg DEX 4 mg Ara-C 36 mg	D1	1	First	Tremors (D3)	15 μg·m^−2^·day^−1^	None, proceeded with close monitoring.	No
2	13, M	2%	MTX 12 mg DEX 4 mg Ara-C 36 mg	D1	2	Second	Tremors (D3)	15 μg·m^−2^·day^−1^	Treated with DEX, mannitol, and sedative. Permanent discontinuation.	Fever (D2)
3	3, M	2%	MTX 12 mg DEX 4 mg Ara-C 36 mg	D1	4	Second	Tremors (D2); seizures lasted for 10 minutes (D4)	15 μg·m^−2^·day^−1^	Treated with DEX, mannitol, and antiepileptic treatment. Permanent discontinuation.	Fever (D2)
4	13, F	<1%	MTX 12 mg DEX 4 mg Ara-C 36 mg	D1	4	Second	Tremors, seizures lasted for 7 minutes (D5)	15 μg·m^−2^·day^−1^	Treated with DEX, mannitol, and antiepileptic treatment. Permanent discontinuation.	Fever (D2)
5	14, M	55%	MTX 12 mg DEX 4 mg Ara-C 36 mg	D28	1	First	Paresthesia (D2); tremors (D9)	9 μg	Treated with steroids and levetiracetam. Brief interruption on Day 3 for concurrent CRS and hyperbilirubinemia.	Fever (D1), hyperbilirubinemia, elevated transaminases, low IgG levels
6	7, M	<1%		N/A	4	Second	Recurrent seizures (D3)	15 μg·m^−2^·day^−1^	Treated with DEX, mannitol, and antiepileptic treatment. Permanent discontinuation.	Fever (D1)
7	11, M	<1%		N/A	1	Second	Paresthesia (D5)	15 μg·m^−2^·day^−1^	None, proceeded with close monitoring. Self-resolved after 4 days.	No
8	6, M	<1%		N/A	1	First	Tremors (D9)	15 μg·m^−2^·day^−1^	None, proceeded with close monitoring.	Fever (D2)

y: years; N/A, not applicable; D, day; M, male; F, female; BLIN, blinatumomab; Ara-C, cytarabine; MTX, methotrexate; DEX: dexamethasone; CRS, cytokine release syndrome.

**Figure 1:**
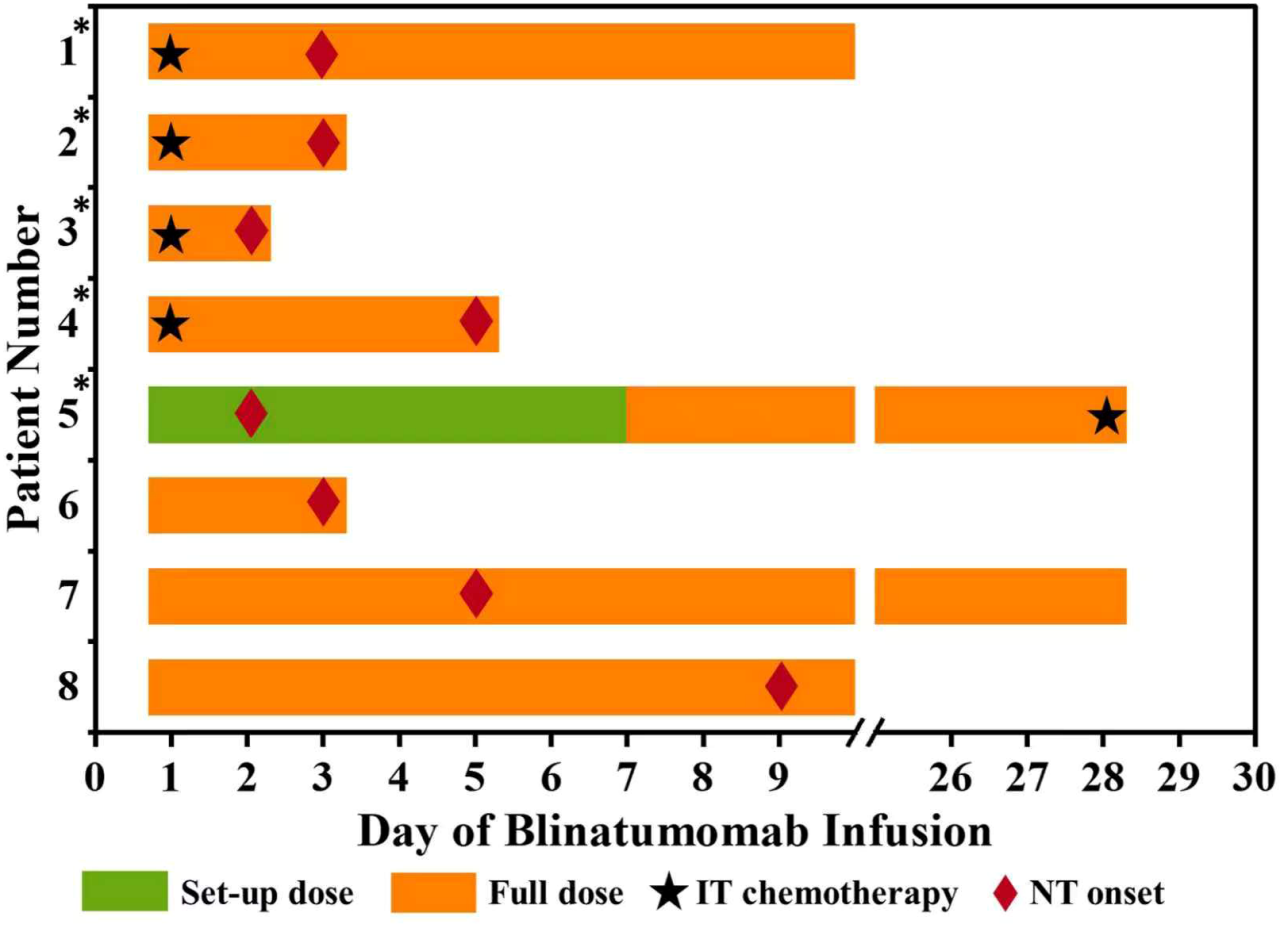
Timing of IT chemotherapy and onset of neurotoxicity. ^*^ Patients receiving IT chemotherapy. IT, intrathecal; NT, neurotoxicity.

In the concurrent IT group, four of the five neurotoxicity cases followed IT chemotherapy administered on Day 1 of blinatumomab. The median interval from IT administration to neurotoxicity onset in these cases was 3 days (IQR: 2.5–4). The median time from initiating blinatumomab treatment to the onset of neurotoxicity was 3 days (IQR: 2–9), and no significant differences were evident between the concurrent IT and control groups (3 [2, 3] days vs. 5 [2, 9] days, p = 0.349). Temporal associations are presented in **Figure 1**.

Patients were treated with one to two cycles of blinatumomab. Neurotoxicity was reported in 4.76% (3/63) of the patients after the first cycle and in 16.67% (5/30) following the second cycle. However, the difference between the two proportions was found to be non-significant on univariable logistic regression, which did identify several variables significantly associated with neurotoxicity: IT administration on Day 1 of blinatumomab (OR = 15.6; 95% CI: 2.96–87.8; p = 0.001), lower CD4+ T-cell levels (OR = 0.03; 95% CI: 0.00–0.50; p = 0.045), higher serum albumin (OR = 1.17; 95% CI: 1.02–1.39; p = 0.042), and higher bone marrow blast percentage (OR = 1.05; 95% CI: 1.01–1.09; p = 0.017). On multivariate analysis, concurrent IT chemotherapy on Day 1 remained a significant independent predictor (OR = 12.5; 95% CI: 1.45–131; p = 0.023) (**Table 3**).

**Table 3:** Univariable and multivariable logistic regression analyses for neurotoxicity.

Characteristic	Univariable analysis			Multivariable analysis		
	OR	95% CI	P-value	OR	95% CI	P-value
Concurrent IT	2.16	0.50, 11.1	0.31			
Age (years)	1.09	0.93, 1.29	0.26			
IT within 2 weeks	2.70	0.59, 12.3	0.18			
IT On D1	15.6	2.96, 87.8	0.001	12.5	1.45, 131	0.023
B cells	0.00	0.00, 0.91	0.27			
T cells	0.52	0.15, 1.22	0.24			
CD4+ T cells	0.03	0.00, 0.50	0.045	0.03	0.00, 0.91	0.11
CD8+ T cells	0.81	0.13, 2.47	0.78			
NK cells	0.49	0.00, 39.8	0.80			
B/T	0.97		0.90			
WBC	1.14	0.87, 1.44	0.27			
Albumin level	1.17	1.02, 1.39	0.042	1.17	0.98, 1.48	0.11
Cycle of blinatumomab	1.93	0.63, 5.34	0.20			
Number of previous IT doses	0.98	0.87, 1.08	0.65			
History of CNS (+) ALL	0.00		>0.99			
Male sex	3.82	0.64, 73.1	0.22			
Bone marrow blasts %	1.05	1.01, 1.09	0.017	1.07	1.01, 1.15	0.054
Relapsed or refractory ALL	0.00		>0.99			
Any grade CRS	0.78	0.18, 4.00	0.74			
Dose escalation	2.20	0.48, 15.6	0.35			

OR, odds ratio; CI, confidence interval; WBC, white blood cell; B/T, B-cell count/T-cell count; CNS, central nervous system; ALL, acute lymphoblastic leukemia; CRS, cytokine release syndrome.

## Discussion

Data on the safety of administering IT chemotherapy concurrently with blinatumomab remain limited, particularly in pediatric populations. While retrospective studies in adults offer conflicting conclusions, no pediatric-specific findings have been published to date. Ngo et al. (3) reported significantly lower neurotoxicity rates with concurrent IT chemotherapy (5.3% vs. 27.2%; p = 0.004), suggesting a potential neuroprotective effect. In contrast, Lee et al. (4) observed higher neurotoxicity rates when IT chemotherapy was given within 24 hours of initiating or during the early phase (first 14 days) of blinatumomab treatment. These conflicting outcomes may be attributable to differences in the timing of IT administration. Notably, Ngo’s study delivered IT chemotherapy at the end of blinatumomab treatment, whereas the cohort of Lee et al. administered IT therapy near the start. Similarly, a case series involving 11 patients with active or prior CNS-positive ALL who received concurrent blinatumomab and IT chemotherapy reported grade 3–4 neurotoxicity in two patients (18%) (5). However, details such as the specific IT regimens used, frequency of lower-grade neurotoxicity, and exact timing of therapies were not disclosed.

Consistent with a prior real-world study that reported a median time of 2 days to the onset of neurological events in pediatric patients (<18 years) receiving blinatumomab (6), our study also observed early neurotoxicity, with a median onset of 3 days (IQR: 2–5), in the overall cohort. Although the difference was non-significant, the incidence of neurotoxicity was twice as high in the concurrent IT group (12%) compared to the controls (6%), suggesting that the IT procedure may have been responsible. Although five of the eight cases of neurotoxicity occurred during the second cycle of blinatumomab treatment, multivariable analysis indicated that this difference in distribution was non-significant. In contrast, neurotoxicity exhibited a clear temporal association with the timing of IT chemotherapy relative to blinatumomab initiation in our study. While concurrent IT administration or IT therapy within 2 weeks of starting blinatumomab did not independently predict neurotoxicity, administration on Day 1 of blinatumomab infusion was significantly associated with neurotoxicity in both univariate and multivariate analyses. Since all patients in our cohort received triple IT therapy (methotrexate, cytarabine, and dexamethasone), we were unable to evaluate the individual contributions of methotrexate or cytarabine to neurotoxicity risk.

Blinatumomab-related neurotoxicity is classified as immune effector cell-associated neurotoxicity syndrome (ICANS) and is a well-documented adverse event associated with CD19-targeted therapies, including chimeric antigen receptor T (CAR-T) cells and bispecific antibodies (7). Although its precise pathophysiology remains uncertain, current models propose a two-step mechanism: first, a B cell-independent redistribution of peripheral T cells involving endothelial adhesion, activation, and perivascular migration; and second, B cell-dependent T-cell activation leading to cytokine release and neuroinflammatory cascades (8). Given the overlap with CRS, corticosteroids remain the cornerstone of ICANS management. Notably, blinatumomab has demonstrated the highest neurotoxicity risk among bispecific T-cell engager antibodies, potentially due to CD19 expression on perivascular mural cells within the brain (9). Theoretical models propose that IT chemotherapy may suppress T-cell activity and mitigate cytokine-driven neurotoxicity. In practice, IT regimens containing steroids (with or without methotrexate or cytarabine) have been used to manage steroid-refractory ICANS in CAR-T therapy recipients (10). Moreover, a case report described secondary prophylactic IT chemotherapy administration before initiating blinatumomab (11). A prospective trial is also underway to evaluate whether IT chemoprophylaxis prior to blinatumomab can prevent neurotoxicity in pediatric ALL (12). These findings support the hypothesis that IT chemotherapy given before or late in the course of blinatumomab infusion, when cytokine levels may be lower, could have neuroprotective effects. Conversely, IT chemotherapy administered at the onset of blinatumomab (as in our study) or during early treatment phases (as in the study of Lee et al.) may exacerbate neurotoxicity due to elevated baseline cytokine levels and increased susceptibility to neuroinflammatory insult.

We observed a neurotoxicity rate of 8.6% (8/93), which is lower than the rates previously reported in adult populations (all-grade, 47%–53%; grade ≥3, 7%–13%) (13, 14). This discrepancy may be explained by the clinical context of our cohort, in which most patients had achieved bone marrow remission prior to starting blinatumomab, in contrast to adult studies, where patients often presented with high disease burden.

In summary, the present findings indicate that concurrent IT chemotherapy administered on Day 1 of blinatumomab therapy is significantly associated with an increased risk of neurotoxicity in pediatric ALL patients. We therefore recommend avoiding IT chemotherapy on the same day as blinatumomab initiation. Given the central importance of IT chemotherapy as a means of managing CNS leukemia, the development of evidence-based guidelines is imperative to ensure that treatment can be timed optimally relative to blinatumomab administration while minimizing the risk of neurotoxicity.

## Data Availability

The datasets presented in this study can be found in online repositories. The names of the repository/repositories and accession number(s) can be found in the article/Supplementary Material.
